# Local resources of polyribosomes and SER promote synapse enlargement and spine clustering after long-term potentiation in adult rat hippocampus

**DOI:** 10.1038/s41598-019-40520-x

**Published:** 2019-03-07

**Authors:** Michael A. Chirillo, Mikayla S. Waters, Laurence F. Lindsey, Jennifer N. Bourne, Kristen M. Harris

**Affiliations:** 1 Center for Learning and Memory, Department of Neuroscience, The University of Texas at Austin, Austin, Texas 78712 USA; 2 Present Address: Fulbright U.S. Scholar Program, University of Belgrade, Studentski trg 1, Belgrade, 11000 Serbia; 3 Present Address: McGovern Medical School in Houston, 6431 Fannin St., Houston, TX 77030 USA; 4 Present Address: Google Seattle, Seattle, Washington 98103 USA; 5 Present Address: Department of Cell and Developmental Biology, University of Colorado, Anschutz Medical Campus, Aurora, Colorado 80045 USA

## Abstract

Synapse clustering facilitates circuit integration, learning, and memory. Long-term potentiation (LTP) of mature neurons produces synapse enlargement balanced by fewer spines, raising the question of how clusters form despite this homeostatic regulation of total synaptic weight. Three-dimensional reconstruction from serial section electron microscopy (3DEM) revealed the shapes and distributions of smooth endoplasmic reticulum (SER) and polyribosomes, subcellular resources important for synapse enlargement and spine outgrowth. Compared to control stimulation, synapses were enlarged two hours after LTP on resource-rich spines containing polyribosomes (4% larger than control) or SER (15% larger). SER in spines shifted from a single tubule to complex spine apparatus after LTP. Negligible synapse enlargement (0.6%) occurred on resource-poor spines lacking SER and polyribosomes. Dendrites were divided into discrete synaptic clusters surrounded by asynaptic segments. Spine density was lowest in clusters having only resource-poor spines, especially following LTP. In contrast, resource-rich spines preserved neighboring resource-poor spines and formed larger clusters with elevated total synaptic weight following LTP. These clusters also had more shaft SER branches, which could sequester cargo locally to support synapse growth and spinogenesis. Thus, resources appear to be redistributed to synaptic clusters with LTP-related synapse enlargement while homeostatic regulation suppressed spine outgrowth in resource-poor synaptic clusters.

## Introduction

In the past decade, much work has revealed that clusters of dendritic spines form a computational unit of synaptic plasticity, learning, and memory^1–15^. LTP is a cellular mechanism of learning and memory known to alter dendritic spines and synapses. In the mature hippocampus, LTP-related synapse enlargement is balanced by stalling spinogenesis^16,17^. In contrast, LTP induced in the developing hippocampus enhances spinogenesis^18^. Live imaging of cultured neuronal dendrites reveals that when potentiation is restricted to one or a few spines, other spines further along the same dendrite shrink or disappear^19,20^. Individual axons that synapse on neighboring spines from the same dendrite produce clusters^15^. However, in many brain regions this axon connectivity-induced clustering is rare, yet spines still appear to form clusters along those dendrites as well^11^. Computational modeling shows that spine clusters can facilitate the spread of synaptic signals throughout the dendritic arbor, and depending on brain region and activation patterns can also facilitate the sharing of resources locally^15,21^. As such, the local events initiated in synaptic clusters could participate in synaptic tagging and capture of subsequent resources that sustain LTP^22–24^. Subcellular resources necessary to maintain LTP are limited and their redistribution to potentiated synapses could preserve spines in clusters undergoing LTP or learning^13^. Redistribution of resources away from non-potentiated synapses would provide a homeostatic mechanism to balance total synaptic weight, measured structurally as summed synaptic surface area along mature dendrites^16^. This homeostatic process is disrupted in many disease states^25,26^. Thus, it is important to learn how spine and synaptic clusters form and under what circumstances they are maintained.

One resource that controls dendritic spine number and synapse size locally is smooth endoplasmic reticulum (SER). SER is a continuous membrane network extending from the soma throughout neuronal processes and into some dendritic spines^27,28^. SER regulates calcium both globally^29^ and locally and provides post-translational modification and trafficking of integral membrane proteins^30^. In dendritic shafts, local regions of SER complexity with high volume or branching retain cargo, such as ion channels and receptors, for enhanced delivery to nearby synapses via ER exit sites^31,32^. In dendritic spines, SER provides compartmentalized regulation of calcium during synaptic plasticity^33–36^. Some large spines contain a spine apparatus, in which folds or cisterns of SER are stacked around dense-staining plates that contain the actin binding protein synaptopodin^37–40^. Eliminating synaptopodin results in loss of the spine apparatus coincident with impaired LTP and learning^40–42^. Immunogold labeling of the spine apparatus reveals molecules involved in the synthesis and post-translational modification of proteins^43,44^. These features suggest that the spine apparatus is a small Golgi outpost, similar to the dynamic Golgi outposts in dendritic shafts^45^. Labeling and imaging of SER-specific molecules has revealed it to be a highly dynamic structure that moves in and out of dendritic spines, in developing, organotypic, and cultured neurons^32,33,36,46^. Similarly, synaptopodin protein has been used as a proxy to track plasticity of putative spine apparatuses in developing or cultured neurons^47,48^. Synaptopodin occurs in other subcellular structures and in developing dendrites prior to the formation of spines or the spine apparatus, suggesting it could also be a marker of dynamics unrelated to the spine apparatus^28,49^. Little is known about the dynamics of SER during synaptic plasticity in the mature brain^30^. The SER enters less than 13% of mature CA1 dendritic spines and forms a spine apparatus in less than 10% of these spines *in vivo*^28,50^. All of these characteristics indicate that three-dimensional reconstruction from serial section electron microscopy (3DEM) is required to determine the structure and location of SER and the spine apparatus.

Local protein synthesis is another resource that can control the outgrowth of dendritic spines and synapse enlargement during LTP and other forms of plasticity^24,51^. Polyribosomes (which are readily identified through 3DEM)^52,53^ provide a conservative estimate of local translation because monosomes (which are not readily identified) are also capable of protein translation^54,55^. Polyribosomes undergo both rapid and sustained changes in frequency, demonstrating the dynamic state of local protein synthesis following LTP and learning^52,56^. However, the role of polyribosomes in local spine and synapse clustering is not known.

Here we used 3DEM to determine whether synaptic clusters are delimited by the availability of SER and polyribosomes in adult hippocampus. We tested whether spines with enlarged synapses collaborated or competed with their neighbors for resources to strengthen local synaptic input after LTP, relative to control stimulation in the same adult hippocampal slices.

## Results

**Figure 1:**
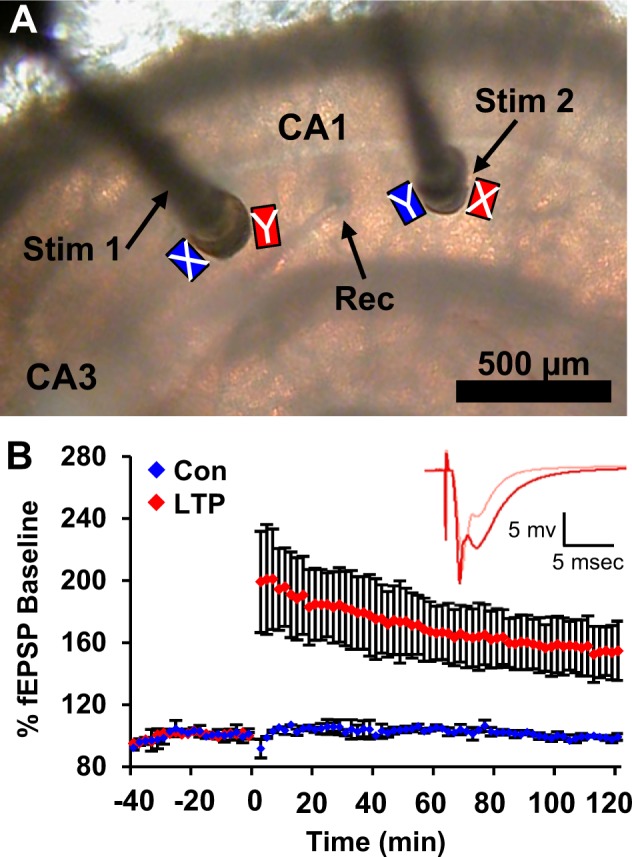
Sampling regions of differential activation in slices with TBS-induced LTP versus control stimulation. (**A**) Acute hippocampal slice in interface chamber with a recording electrode (Rec) located in the middle of *stratum radiatum* of hippocampal area CA1 between two stimulating electrodes (Stim 1 and Stim 2) separated by >600 µm. Rectangles indicate regions that samples were collected for 3DEM near the stimulating electrodes. LTP was induced with TBS and a stimulus intensity set above population spike threshold to maximize the probability that most (if not all) synapses within the field of the stimulating electrode were potentiated. In one experiment (X), control stimulation was delivered to Stim 1 (blue X) and TBS was delivered to Stim 2 (red X). In the other experiment (Y), TBS was delivered to Stim 1 (red Y) and control stimulation was delivered to Stim 2 (blue Y). In this way, the experiments were counterbalanced for which sites (CA3 or subicular side of CA1) obtained control (blue) or LTP-inducing (red) stimulation. (**B**) Changes in the slope of the fEPSP (%) relative to baseline test pulses following induction of LTP at time 0 (red), or from control stimulation alone (blue) (n = 2, mean ± SD of responses from two slices from different animals. Inset shows example waveforms from responses to the stimulating electrode before (pink) and 120 min after (red) TBS (adapted from Bourne and Harris, 2011^16^).

These investigations were performed 2 hours following the induction of LTP. Slices were prepared from the middle of adult rat hippocampus. Two stimulating electrodes were placed on either side of a recording electrode in *stratum radiatum* of area CA1 to demonstrate input specificity of control and potentiated synapses (Fig. 1A)^4^. To induce LTP, theta-burst stimulation (TBS) was delivered to one stimulating electrode at an intensity and pattern that produces multiple action potentials, engages multiple molecular mechanisms, and saturates LTP, thus maximizing the likelihood of observing long-lasting ultrastructural outcomes^57^. Both the control and TBS electrodes received the same total number of test pulses at a rate of 1 test pulse per 2 minutes throughout the experiment (Fig. 1B). The site of LTP induction (CA3 or subicular side of CA1) was alternated between experiments. At 2 hours following TBS, the slices were rapidly fixed, processed, imaged, and analyzed using unbiased approaches of 3DEM with experimenters masked as to condition (see Methods).

### Impact of spine SER on synapse enlargement with LTP

**Figure 2:**
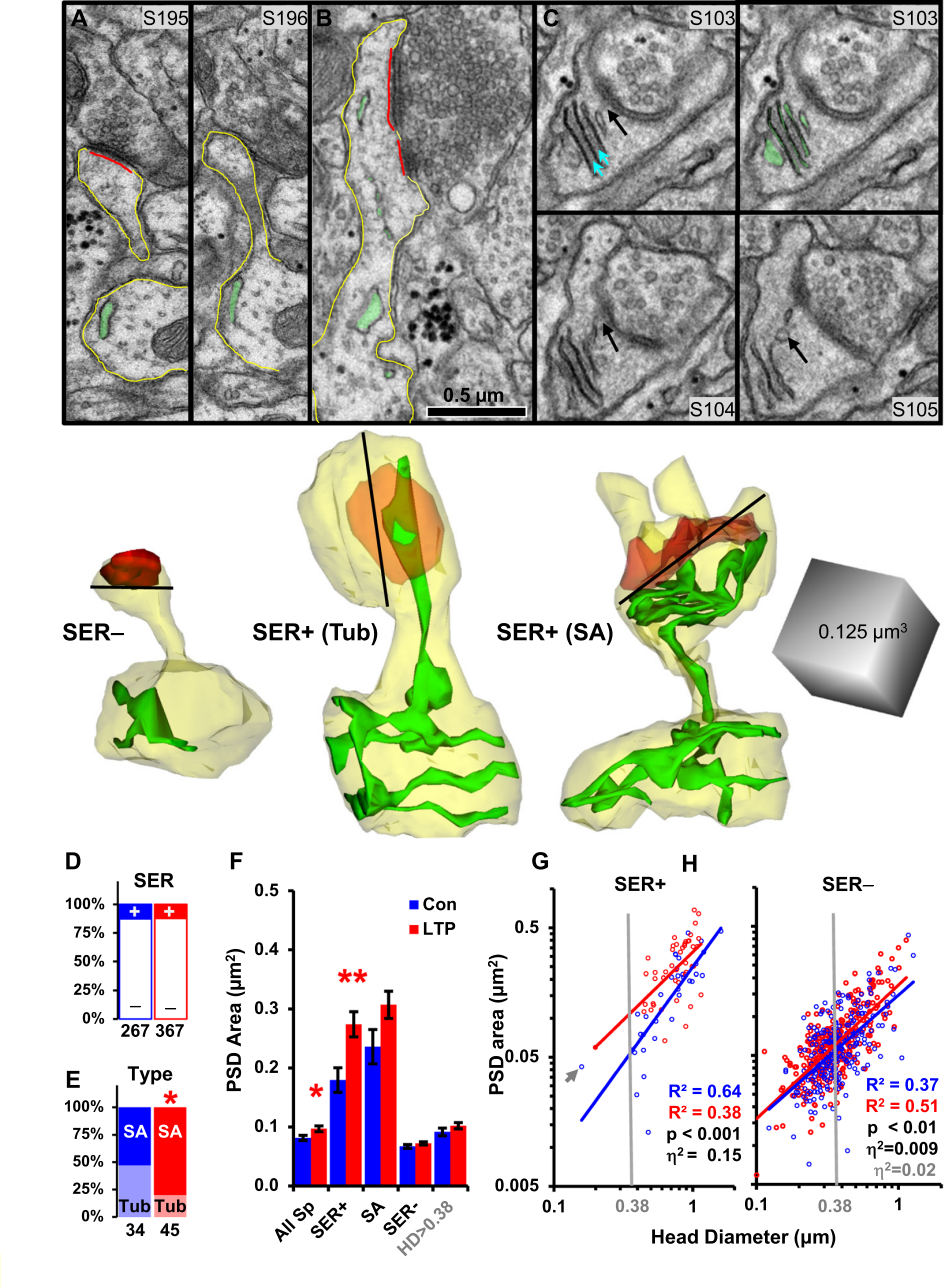
Spine SER forms an apparatus and boosts synapse enlargement following LTP. Serial section images (S#) and 3D reconstructions of spines (**A**) without SER (SER−) from LTP, (**B**) with single SER tubule (SER+ (Tub)) from control, and (**C**) with apparatus (SER+ (SA)) from LTP. The SA has dense material (S103, blue arrows) directed to the postsynaptic density (PSD; S103-104, black arrows), and a vesicle (S105, black arrow). Spine head diameters were measured at widest point (black line in 3Ds). (SER (green), spine (yellow), PSD (red). Scale bar in B is for all EMs and matches the edge of the cube, which is for all 3Ds). (**D**) Percentage of SER+ spines in control (12.7%, n = 267) and LTP (12.2%, n = 367) conditions versus SER− spines (χ^2^ = 0.03, p = 0.86). (**E**) Percentages of SER+ spines with an apparatus (SA) versus tubule (T) in LTP (80%, n = 45) and control (47%, n = 34) conditions (χ^2^ = 6.6, *p = 0.01). (**F**) Mean PSD areas differed between SER+ vs SER− in control (ANOVA, F_(1, 265)_ = 56, p < 0.0001) or LTP (ANOVA, F_(1,365)_ = 222, p < 0.0001) conditions, between control (0.081 ± 0.004 µm^2^) vs LTP (0.096 ± 0.005 µm^2^) for all spines (All Sp, hnANOVA, F_(1,616)_ = 4.40, *p = 0.036, η^2^ = 0.0014), and between LTP vs Control for SER+ spines (hnANOVA, F_(1,61)_ = 5.7, **p = 0.020, η^2^ = 0.070). SA+ spines had largest PSD areas in both control (0.24 ± 0.3) and LTP (0.31 ± 0.3µm^2^) conditions but did not reach significance between conditions (ANOVA, F_(1,50)_ = 3.1, p = 0.07). The PSD areas on SER− spines did not differ overall (hnANOVA, F_(1,537)_ = 2.4, p = 0.13) or for head diameters >0.15 µm (F_(1,533)_ = 3.1, p = 0.09) or >0.38 µm (hnANOVA, F_(1,207)_ = 2.0, p = 0.16). (**G**,**H**) The relationship between PSD area and head diameter was well-correlated in both conditions (p < 0.001) and had homogeneity of slopes across LTP and control conditions for both SER+ and SER− spines. (Gray arrow is smallest SER+ spine; vertical gray line is smallest SA+ spine). (**G**) The PSD areas on SER+ spines were greater in LTP than control conditions across head diameters (ANCOVA, F_(1,76)_ = 13.7, p = 0.00041; η^2^ = 0.15, i.e. 15%). (**H**) For SER− spines, the statistically significant effect (ANCOVA, F_(1,552)_ = 8.7, p = 0.0034) was weak (η^2^ = 0.009, 0.9%) and was accounted for by spine head diameters >0.38 µm (ANCOVA, F_(1,222)_ = 7.8, p = 0.006, η^2^ = 0.022, 2.2%) compared to insignificant effect between LTP and control conditions for head diameters <0.38 µm (ANCOVA, F_(1,327)_ = 2.19, p = 0.14).

We hypothesized that synapses on spines containing SER would be larger than those without, because they would have ready access to this crucial resource. Synapse size was measured as the area of the postsynaptic density (PSD) along the surface of the dendrite membrane. The SER was identified as irregularly shaped, membranous cisternae with a clear lumen (Fig. 2). Most dendritic spines contained no SER (Fig. 2A). When the SER entered dendritic spines, it formed either a single tubule (Fig. 2B) or a spine apparatus comprised of stacked folds of SER, dense plates, and vesicles (Fig. 2C). The fraction of dendritic spines containing some form of SER was unaffected by LTP (Fig. 2D). However, by 2 hours following the induction of LTP, the ratio of spines with a spine apparatus to spines with a simple SER tubule was markedly increased relative to control stimulation (Fig. 2E). Spines with SER of either form had the largest synapses in both conditions (Fig. 2F). However, only spines with SER showed significantly larger synapses in the LTP relative to the control condition (Fig. 2F).

These findings raised the question of whether spines with SER accommodated more synaptic growth simply because they were larger than the spines without SER. Spine head diameters did not differ significantly across the LTP and control conditions for spines with or without SER (Fig. 2G,H). However, when controlling for head diameter, the spines with SER showed a 15% enlargement in synapse area following LTP compared to control stimulation (Fig. 2G). In contrast, synapses on spines without SER were only 0.9% larger in the LTP versus control condition across spines of all head sizes (Fig. 2H).

All spines containing a spine apparatus had head diameters greater than 0.38 µm (gray vertical line, Fig. 2G). The smallest spine containing a single tubule of SER in the control condition had a head diameter of 0.15 µm (gray arrow, Fig. 2G). Restricting the analysis to spines with head diameters greater than 0.38 µm showed spines without SER had synapses 2.2% larger in the LTP compared to control. This outcome was still much less than the 15% difference after LTP for spines of similar size with SER (Fig. 2F–H). Spines without SER and head diameters less than or equal to 0.38 µm showed no significant differences in PSD area between LTP and control conditions. Overall these findings suggest that, by 2 hours after TBS-induced LTP, tubules of SER elaborated to form a spine apparatus in some spines and synapses on spines containing SER underwent the greatest enlargement.

### Impact of spine polyribosomes on synapse enlargement after LTP

**Figure 3:**
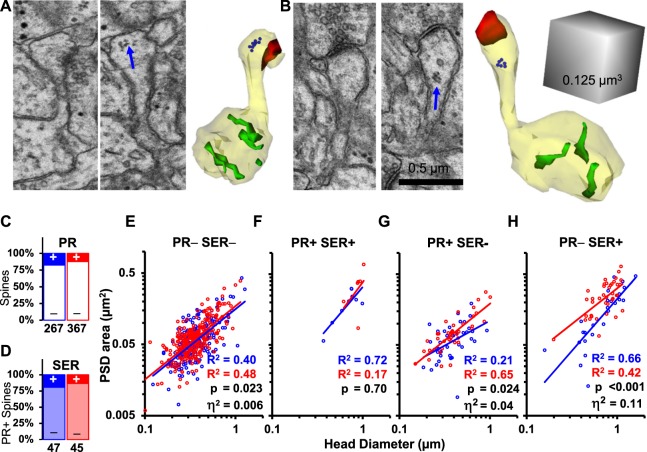
Distinct effects of polyribosomes and SER on synapse size. Serial electron micrographs and 3D reconstructions illustrate polyribosomes (blue arrows) in the heads of spines from the (**A**) control and (**B**) LTP conditions. (3Ds – spine and dendrite (yellow), PSD areas (red), SER (green), each ribosome in the polyribosome (blue 20 nm sphere)). (**C**) In the control condition (blue), 17.6% of spines contained polyribosomes (PR) while in the LTP condition (red) 12.3% of spines contained PR (χ^2^ = 3.6, p = 0.059). (**D**) Of the PR+ spines, 19.2% also contained SER in control, and 13.3% also contained SER in the LTP condition (χ^2^ = 0.6, p = 0.45).There was a strong positive correlation between log-normalized PSD areas and spine head diameters across spines with different sets of resources in the control and LTP conditions. Analysis of covariance accounting for head diameters revealed: (**E**) slightly larger PSD areas on PR− SER− spines in the LTP vs control conditions (F_(1,475)_ = 5.22, n = 478); (**F**) PR+ SER+ spines were rare and no statistically significant differences were detected in PSD areas between the LTP and control conditions (F_(1,12)_ = 0.16, n = 15); (**G**) PR+ SER− spines were significantly larger in PSD area in the LTP condition (F_(1,74)_ = 5.3, n = 77); and (**H**) PR− SER+ spines had the most enlargement of PSD area in the LTP vs control condition (F_(1,61)_ = 16.6, n = 64). Correlations for each condition, p values for the LTP vs control comparisons, and effect sizes (η^2^) of condition are presented in the lower corner of each graph.

Prior work revealed that overall the frequency of polyribosomes was decreased in spines by 2 hours following the induction of LTP by TBS; however, spines that retained polyribosomes had larger synapses than those without polyribosomes^16^. Independent or combined effects of SER and polyribosomes were not considered, nor was the analysis controlled for spine head dimensions. Here, we analyzed all spines for the presence of polyribosomes with or without SER. Polyribosomes were identified in the spines as clusters of 3 or more ribosomes in the control (Fig. 3A) or LTP condition (Fig. 3B). Polyribosomes were found in ~18% of spines in the control condition and ~12% of spines in the LTP condition. This difference was not statistically significant (Fig. 3C). Polyribosomes and SER occurred together in the same spine in 10% of control spines and 7% of spines in the LTP condition (Fig. 3D).

When controlling for head diameter, we found that synapses on spines lacking both polyribosomes and SER were only 0.6% larger after LTP relative to control conditions (Fig. 3E). Spines containing both polyribosomes and SER had large synapses with large heads in both conditions (Fig. 3F). Spines with polyribosomes but no SER were 4% larger after LTP relative to control (Fig. 3G). Spines with SER but no polyribosomes were almost 3 times larger after LTP (11%) than those with polyribosomes but no SER (Fig. 3H). These findings suggest that subtle growth in synapse area occurred on large spines without SER or spines containing polyribosomes only, while the presence of SER substantially boosted synapse enlargement at 2 hours after the induction of LTP.

### Delineating synaptic clusters

**Figure 4:**
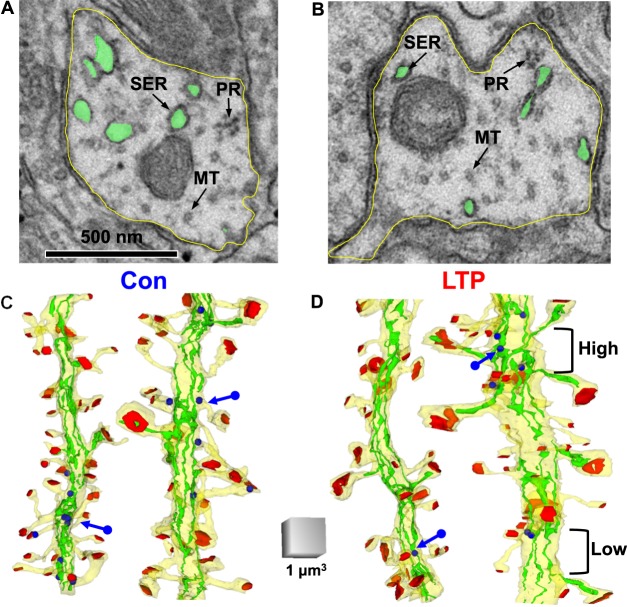
Nonuniform density of spines. The electron micrographs illustrate single sections through the shaft of similar caliber dendrites from the (**A**) control and (**B**) LTP conditions. The dendrite membrane (yellow) with SER (green), representative microtubules (MT, arrow), and polyribosomes (PR, arrow) are indicated in the dendritic shafts. The 3D reconstructions of dendrites (yellow) from (**C**) Control and (**D**) LTP conditions include PSD areas (red), polyribosomes (blue arrows and spheres), and SER (green). Regions of high and low spine densities occur in patches along the dendritic segments.

Prior work showed that synapse enlargement on some spines was perfectly balanced by a lower spine density at two hours following induction of LTP by TBS^16^. Additional analyses suggested that the lower density resulted from an LTP-related stalling of spine formation, which normally occurs in adult hippocampal slices when 3 hours of recovery plus 3 hours of control stimulation returns spine density to *in vivo* levels^17^. We wanted to know whether local resources influenced this process. We quantified polyribosomes, microtubules, and SER through serial sections of the dendritic segments (Fig. 4A–D). Visual inspection revealed regions of high versus low spine density along the 3D reconstructions of dendrites, especially in the LTP condition (Fig. 4D).

**Figure 5:**
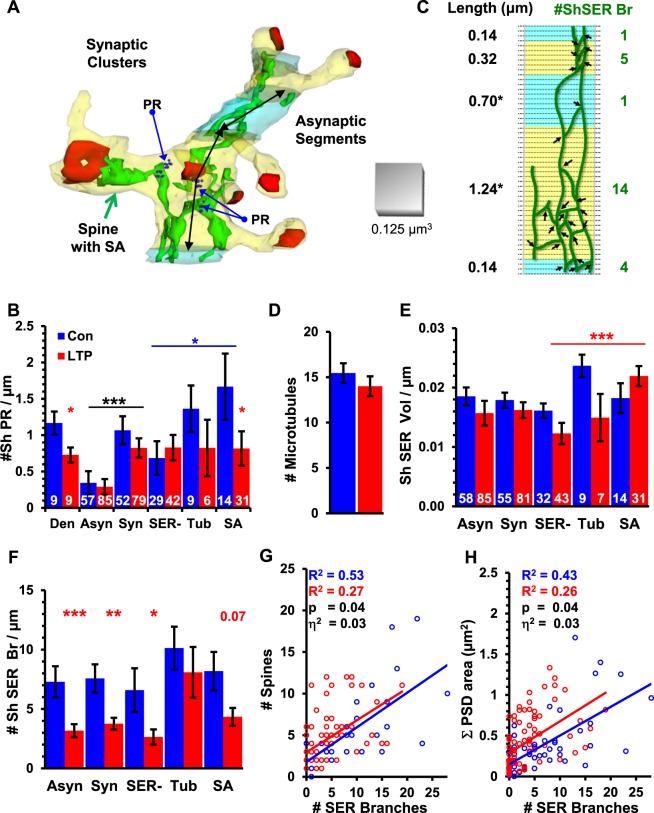
Effect of LTP on local polyribosomes, SER, spine density, and total synaptic weight. (**A**) Synaptic clusters (yellow) surrounded by asynaptic segments (light blue) showing shaft SER extending into SA (green) and polyribosomes (PR) at that spine base and in the dendritic shaft (1 dark blue sphere per ribosome). Double-headed black arrows show oblique segment lengths. (**B**) Density of PR in dendrites (Den) was significantly lower in LTP vs control (ANOVA, F_(1,16)_ = 5.4, p = 0.03, η^2^ = 0.25, red asterisk). Asynaptic segments (Asyn) had fewer PR than synaptic clusters (Syn), (ANOVA, F_(1,275)_ = 18.8, p = 0.00002, η^2^ = 0.064, black asterisks), not differing by condition. Control SA or Tub clusters had more PR than SER− clusters (LSD, p = 0.02, blue asterisk). Control SA clusters had more PR than LTP (LSD, p = 0.04, red asterisk). (Six outliers were excluded with inflated PR densities (>6 PR/µm), due to short segment lengths (<0.4 µm): 1 Asyn, 4 SER− control, 1 Tub LTP). (**C**) Diagram SER branch points (arrows, #ShSER Br, green numbers) summed across lengths (black numbers). (EM sections, dotted lines). (**D**) Number of dendritic microtubules (MT) did not differ significantly by condition (ANOVA F_(1,14)_ = 1.07, p = 0.32, n = 18). (**E**) Normalized shaft SER volume did not differ significantly between Asyn and Syn (ANOVA, F_(1,275)_ = 0.13, p = 0.71). For LTP, shaft SER volume was greater in SA than SER− clusters (LSD, p = 0.001). LTP did not differ from Control for Syn (ANOVA F_(1,135)_ = 2.7, p = 0.07), SER− (LSD, p = 0.11), Tub (LSD, p = 0.09), or SA (LSD, p = 0.26) clusters. (White numbers are total clusters by category for (**E**,**F**). (**F**) Shaft SER branch density was greater overall in control (7.4 ± 0.9/µm) than LTP (3.5 ± 0.4/µm) (ANOVA, F_(1,277)_ = 21.3, p < 0.0001, η^2^ = 0.07), Asyn (LSD, p < 0.0007), and Syn (LSD, p < 0.004). Syn shaft SER branch density was higher in control vs LTP (ANOVA, F_(1,135)_ = 5.5, p = 0.02, η^2^ = 0.03), reaching statistical significance only for SER− clusters (LSD, p = 0.010). (**G**) Spine number correlated with shaft SER branches was greater across Syn in LTP than control (p < 0.0001 for both correlations, ANCOVA, F_(1,128)_ = 4.44, p = 0.03, η^2^ = 0.03). (**H**) The summed synaptic area was also correlated with shaft SER branches (p < 0.0001) and greater across Syn clusters in the LTP condition (ANCOVA, F_(1,128)_ = 4.5, p = 0.03, η^2^ = 0.03). (SA, Tub, and SER- refer to clusters that had spines with SA, Tub or for which all spines lacked SER, respectively).

We divided the reconstructed dendrites into discrete synaptic clusters surrounded by asynaptic dendritic segments (Fig. 5A). Synaptic clusters had spine origins and/or shaft synapses along their lengths. To qualify as an asynaptic segment, there had to be no spine origins or synapses for at least 120 nm. This length was chosen because it was twice the length of the smallest spine origin. Some spine and shaft synapses were subsequently excluded if their cluster was not surrounded by asynaptic segments, which would make the end of the synaptic cluster ambiguous. Where the dendrites were in perfect cross-section, the lengths equaled section number times section thickness. Where the dendrites ran obliquely to the plane of section, the lengths were measured down the center of the dendritic shaft.

### Polyribosome redistribution among asynaptic segments and synaptic clusters after LTP

Overall, the frequency of polyribosomes in dendritic shafts and spines was 25% lower at 2 hours following LTP (Fig. 5B)^16^. Polyribosomes were less frequent in asynaptic segments than synaptic clusters (Fig. 5B), as a substantial fraction of polyribosomes occurred in dendritic spines. This differential distribution between asynaptic and synaptic clusters was not altered by TBS-induced LTP. Under control conditions, polyribosomes were highest in synaptic clusters having a spine apparatus and polyribosomes were reduced specifically in spine apparatus-containing clusters in LTP relative to the control conditions (Fig. 5B). As indicated above, spines containing SER formed a spine apparatus and synapses on these spines were largest following LTP.

### Shaft SER redistribution among asynaptic segments and synaptic clusters after LTP

Cargo exit sites from SER occur more frequently in dendritic shafts with more SER volume or branches^31,58^. In these regions, trafficking is slowed and cargo accumulates in support of higher local spine density^31^. Here we tested whether there was a detectable difference after LTP in the volume or branching of SER in the dendritic shaft and in the relationship of the spine apparatus, synapse size, spine density, and total synaptic weight in spine clusters. The number of SER branches in the dendritic shaft was summed across each synaptic cluster or asynaptic segment (Fig. 5C). When SER is bound to microtubules in the dendritic shaft, it elongates and has fewer branch points and ER exit sites^31^. The number of microtubules per dendrite was not significantly different across control and LTP conditions (Fig. 5D) and thus there was similar access to SER binding sites in each condition.

The mean volume of shaft SER was not altered following LTP relative to control stimulation in asynaptic segments or synaptic clusters (Fig. 5E). However, synaptic clusters containing one or more spines with a spine apparatus retained more shaft SER volume than other synaptic clusters in the LTP condition (Fig. 5E). In contrast, the number of shaft SER branches was markedly reduced in both asynaptic segments and synaptic clusters following LTP compared to control (Fig. 5F). Among synaptic clusters, this LTP-related reduction did not reach significance in those clusters that had spines containing SER as a tubule or spine apparatus (Fig. 5F). A direct comparison of spine number (Fig. 5G) versus the number of shaft SER branches in a cluster showed a drop in the correlations following LTP. The total synaptic weight was computed by summing the surface areas across all synapses in a cluster and was compared to the number of shaft SER branches in the cluster; this correlation also showed a subtle reduction after LTP compared to control stimulation (Fig. 5H). Analysis of covariance showed that these SER branches supported 3% more spines and 3% more synaptic weight following LTP compared to control stimulation (Fig. 5G,H). Together, these results suggest that local SER resources are retained in regions of the dendritic shaft where synapses were larger following LTP.

### Spine density highest, and preserved after LTP, in resource-rich clusters

**Figure 6:**
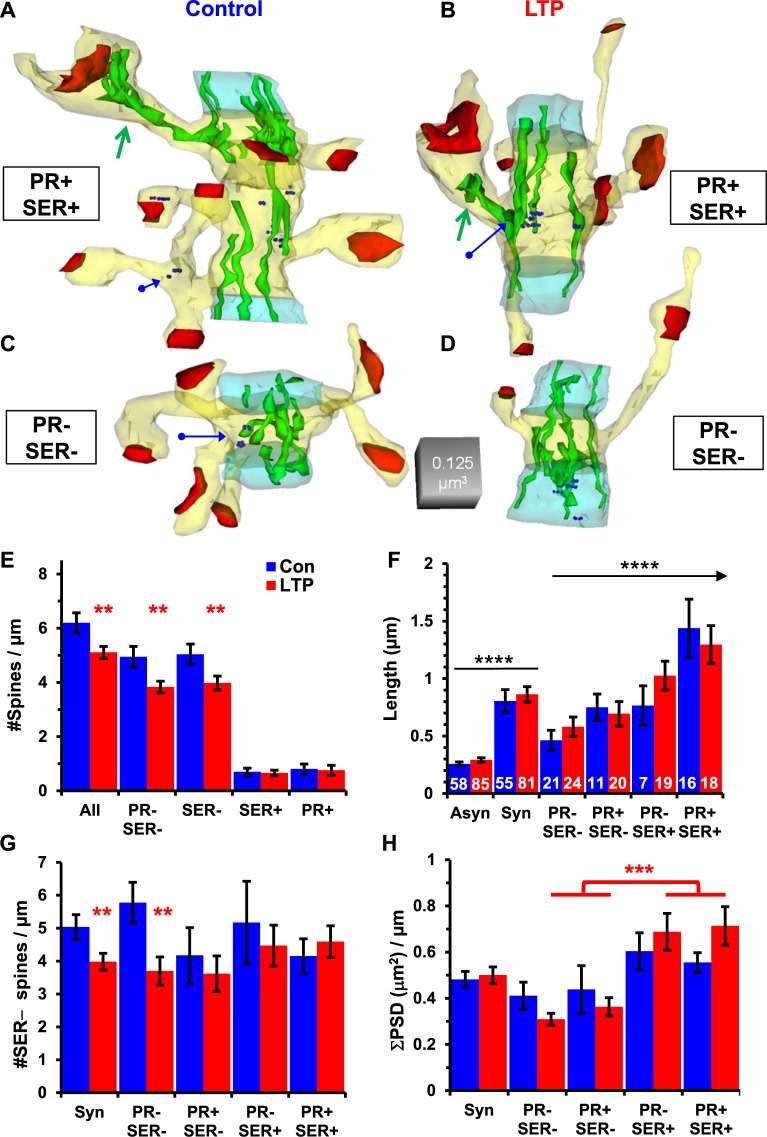
Preservation of resource-poor spines in synaptic clusters with resource-rich and enlarged spines. Synaptic clusters from (**A**) control or (**B**) LTP with at least one SER+ spine, and in these cases, PR+ spines. (**C**) Control and (**D**) LTP synaptic clusters with SER− spines only. (**E**) Spine density was greater in clusters from Control (6.20 ± 0.37/µm) than LTP (5.11 ± 0.22/µm) conditions (ANOVA, F_(1,132)_ = 6.10, p = 0.015, η^2^ = 0.04). The density of PR− SER− spines was greater in Control (4.94 ± 0.4/µm) than LTP (3.83 ± 0.22/µm) conditions (ANOVA, F_(1,132)_ = 6.0, p = 0.016, η^2^ = 0.04) and SER− spines with or without PR (F_(1,132)_ = 5.60, p = 0.019, η^2^ = 0.041). The density of SER+ or PR+ spines was unchanged across conditions (SER+, ANOVA F_(1,132)_ = 0.08, p = 0.78; PR+ F_(1,132)_ = 0.08, p = 0.78). (**F**) Asynaptic segment lengths were comparable across Control (0.25 ± 0.02 µm) and LTP (0.29 ± 0.02 µm) conditions (ANOVA F_(1,139)_ = 1.1, p = 0.2), as were synaptic cluster lengths for Control (0.8 ± 0.1 µm) and LTP (0.86 ± 0.07 µm) conditions (ANOVA F_(1,139)_ = 1.1, p = 0.2). In both conditions, synaptic clusters were longer than asynaptic clusters (F_(1,277)_ = 98, p < 0.001, η^2^ = 0.35). Synaptic clusters with SER+ and PR+ spines (PR+ SER+) were longer in both conditions than PR− SER+ clusters (LSD, p < 0.05), PR+ SER− clusters (LSD, p < 0.01), and PR− SER− clusters (LSD, p < 0.001). (White numbers are the total number of segments or clusters in each category for **F**–**H**). (**G**) PR− SER− synaptic clusters had fewer SER− spines in the LTP than control condition (F_(1,73)_ = 6.08, p = 0.016, η^2^ = 0.077); no other category reached statistical significance. (**H**) Summed synaptic areas, normalized for segment length, were not statistically different between control and LTP conditions (ANOVA, F_(1,135)_ = 0.1, p = 0.75), and none of the post-hoc comparisons across the control synaptic clusters differed significantly. In the LTP condition, the summed synaptic areas were greater in the PR− SER+ and the PR+ SER+ clusters than either of the SER− synaptic clusters (LSD, p < 0.001, red asterisks).

Spines containing polyribosomes or SER had greater synapse enlargement in LTP compared to control conditions than spines lacking these resources. Therefore, we wanted to know whether spine density was compromised or preserved in synaptic clusters with differential synaptic growth and resources following LTP. Synaptic clusters were delineated as having at least one spine with SER (Fig. 6A,B) versus those lacking spines containing SER (Fig. 6C,D). We also tracked whether there were polyribosomes in any of the spines of a synaptic cluster.

Spine density was lower across all synaptic clusters in the LTP condition (Fig. 6E). This lower density was accounted for by spines lacking SER (with or without polyribosomes), whereas the density of spines with SER or with polyribosomes was unchanged in synaptic clusters between LTP and control conditions (Fig. 6E). Asynaptic segments were shorter than synaptic clusters in both control and LTP conditions (Fig. 6F). Synaptic clusters having spines with polyribosomes and/or SER were longer than those without these spine resources (Fig. 6F). This length differential did not differ significantly between the control and LTP conditions, hence LTP-altered spine densities were not simply due to changes in cluster length.

Since only spines lacking SER were reduced in the LTP condition, we considered the effect of neighboring resource-rich spines on those lacking SER in the same clusters (Fig. 6G). In synaptic clusters having no spines with polyribosomes or SER, the density of spines was significantly reduced following LTP compared to controls (Fig. 6G). Synaptic clusters with one or more spines containing polyribosomes or SER showed no difference in the density of neighboring spines lacking SER across control and LTP conditions (Fig. 6G). This finding suggests that only resource-poor synaptic clusters have a reduced spine density following LTP compared to control stimulation.

In prior work, the summed synaptic weight along dendritic segments including both synaptic and asynaptic segments was unchanged in the LTP compared to the control condition^16^. Here, the summed synaptic weight across the synaptic clusters was also unchanged between LTP and control conditions (Fig. 6H). Furthermore, the summed synaptic weight was greatest in synaptic clusters with resource-rich spines that contained SER (Fig. 6H). This effect was balanced by the lower total synaptic weights in the clusters lacking resource-rich spines with SER, which was lowest in the LTP condition (Fig. 6H).

## Discussion

Delineation of synaptic clusters revealed precise relationships between local resources of polyribosomes and SER that affect synapse size and spine density in both conditions. About 12–13% of dendritic spines had SER in both conditions. However, most of the spine SER shifted from a single tubule in the control condition to an elaborate spine apparatus after inducing LTP with TBS. Synapse enlargement was greatest on spines with SER (15%), less on spines containing only polyribosomes (4%), and negligible on spines lacking both resources (0.6%) in the LTP condition relative to control stimulation. Resource-poor spines of comparable size to resource-rich spines showed less synapse enlargement (2%) with LTP. Thus, LTP-related synapse enlargement relied on local resources, not just spine size. Polyribosome frequency was highest in synaptic clusters with resource-rich spines under control conditions. After LTP, polyribosomes were reduced specifically in synaptic clusters that had a spine apparatus, suggesting these polyribosomes had been used for local translation during synapse enlargement. Total synaptic weight, measured as the summed PSD surface area per cluster length, was balanced across LTP and control conditions. Nevertheless, following LTP, resource-rich synaptic clusters had greater total synaptic weight than resource-poor clusters. These outcomes suggest that synapses on spines in resource-poor clusters were not available or ready for potentiation and, further, that these spines were susceptible to and maybe even targeted for removal after the induction of LTP^22–24,59^. Alternatively, ongoing spine turnover and outgrowth may be stalled in the resource-poor clusters as part of the homeostatic balance following LTP^17^.

Prior work showed that dendritic polyribosomes were elevated at 5 minutes after TBS but their abundance declined markedly by 2 hours^16^. Here we showed that this reduction in polyribosomes was specific to resource-rich synaptic clusters that had a spine apparatus. If local translation of mRNAs produced cytoplasmic proteins needed for synapse enlargement, then disassembly of polyribosomes could reflect completion of that process before 2 hours. In contrast, when LTP was induced by tetanic stimulation (3 times 1 second of 100 Hz), polyribosomes remained elevated in dendritic spines for 2 hours^60^. In that study, slices were equilibrated for about 1 hour prior to experimentation, as was commonly done in the past. Later we learned that at least 3 hours are required for spine recovery from slicing^61^. The data reported in the present study are from slices that had been equilibrated for at least 3 hours prior to experimentation. In the prior study, the ongoing recovery from slicing could have influenced the timing or magnitude of local protein synthesis and SER response to LTP^22,61^. In addition, the TBS used here produces many action potentials and engages multiple molecular mechanisms. Tetanic stimulation, on the other hand, produces only one or two action potentials, which could limit molecular mechanisms engaged, mRNA translation, or ribosomal disassembly^24,54^.

SER contributes to the regulation of calcium dynamics directly, or through its interaction with scaffolding and other proteins^35,62–66^. Elevations in calcium can be localized within spines or spread through the dendritic shaft^34,62^, ultimately propagating to the nucleus where calcium transients influence gene transcription^67^. In the hippocampus, ryanodine receptors (RyR) localize to SER in dendritic spines^68^ and respond to calcium entering through ionotropic glutamate receptors and voltage-gated calcium channels^62^. RyR activation results in calcium-mediated calcium release that amplifies an otherwise weak signal^63,69–72^. Inositol trisphosphate receptors (IP_3_Rs), on the other hand, are localized to SER in the dendritic shaft^64,68^ and are activated by calcium and IP_3_^62^. Hotspots of IP_3_Rs occur along SER in CA1 dendrites where SER is more elaborate. Furthermore, calcium released from SER via IP_3_R activation during LTP is involved in coordinating plasticity among synaptic sites along dendrites^73–75^. Thus, SER elaboration in and at the bases of spines during LTP could enhance local calcium signaling and serve as a potentiating signal to sustain and enlarge their synapses^76,77^. In contrast, where dendritic shaft SER comprises simple tubules, less calcium would be released and phosphatases would be more likely to be activated^78,79^, possibly leading to local spine loss.

Whether the spine apparatus formed in response to a local burst in membrane-associated protein synthesis during LTP remains to be determined^43^, partly because rough endoplasmic reticulum could not be identified unequivocally in this material. However, since the spine apparatus has immuno-reactive markers for proteins specific to the Golgi apparatus, it might provide post-translational modification of membrane proteins in spines following LTP^44,45,64,80,81^. Synaptopodin is an essential component of the mature spine apparatus^45^. Live-imaging of cultured neurons shows spines containing synaptopodin have larger AMPA receptor-mediated responses from RyR-triggered calcium release^47^. Two-photon microscopy reveals synaptic depression is also regulated by IP_3_-dependent mobilization of calcium at large spines associated with synaptopodin^33^. Thus, the LTP-related elaboration of the spine apparatus in mature dendritic spines could enhance both the regulation of intra-spine calcium and the post-translational modification and trafficking of proteins at enlarged spine synapses.

Local delivery of SER cargo is greatest where SER structure is most complex, because increased geometric complexity results in slower trafficking of membrane and proteins and forms ER exit sites^31^. In perfusion-fixed hippocampus, shaft SER volume and branching was greater in portions of the dendrite with more or larger dendritic spines^28,31^. Similarly, under the control conditions reported here, shaft SER volume and branching was greatest in synaptic clusters where total synaptic input was highest. These relationships were retained 2 hours after LTP in synaptic clusters that had resource-rich spines. However, shaft SER branching was markedly decreased in asynaptic regions as well as in synaptic clusters lacking resource-rich spines after LTP. These findings suggest that shaft SER membrane was redirected from asynaptic regions and clusters with low spine density to synaptic clusters where spines were preserved, and synapses were enlarged. Several molecular mechanisms could be triggered that would link LTP to elaboration of the spine apparatus and local reorganization of dendritic shaft SER. Protein kinase C-mediated phosphorylation of CLIMP-63, an integral membrane protein of SER, causes SER to dissociate from microtubules and become more elaborate^31,82^. CLIMP-63 could also be phosphorylated by other products of LTP such as Casein kinase II (CKII)^82–84^. Further along the dendrite, away from activated spines, the dephosphorylation of CLIMP-63 causes SER to associate with microtubules and become more tubular^31,82^. This simplification of SER would enhance movement of proteins and other cargo away from less active synaptic clusters, thereby stalling the formation of new spines or leading to the elimination of weak spines^16^.

How these results will generalize to other developmental stages, plasticity paradigms, or brain regions remains to be determined. Glutamate uncaging^20^, or release from cadmium block of extracellular calcium^19^, at multiple spines on cultured hippocampal neurons produced LTP, and resulted in shrinkage or loss of one or a few spines elsewhere along the dendrite. Adult CA1 dendrites in our acute slices have a greater spine density (3–4 spines/µm of the full segment including both synaptic and asynaptic regions)^16^ than CA1 dendrites in the dissociated hippocampal cultures (~1 spine/µm)^19,20^. In the present configuration, TBS should activate most if not all axons in the field. Hence, it is not surprising that these preparation- and paradigm-related differences alter the magnitude of homeostatic balance in spine density after LTP. Prior work also showed that 3 hours of recovery plus 3 hours of control stimulation were needed to return spine density in hippocampal acute slices up to *in vivo* levels for both P15 and adult ages (P55–75)^17^. Nevertheless, even following the requisite slice recovery (and same TBS induction paradigm) contrasting outcomes resulted between these ages, with spinogenesis at P15 but synapse enlargement and compensatory spine reduction in adults^16–18^. The total number of test pulses was the same in both ages and the control stimulation and TBS were delivered in the same slice. Therefore, the effects of age on LTP-related synaptic plasticity were not due to differences in paradigms. Instead the age-related differences in response to LTP likely reflect different stages in synaptogenesis, where P15 dendrites have reached less than 50% of the adult spine density^85^.

Recent findings show that synapse clustering on dendrites can be driven by a single presynaptic axon, which overcomes the electrotonic disadvantage of being located in the distal dendritic tuft of hippocampal CA1 pyramidal cells^15^. However, axonal branches from the same neuron do not form spine clusters on most of the target dendrites, especially in the more proximal CA1 dendritic locations studied here^11,15^. In fact, same dendrite/same axon pairing between synapses is quite rare in the middle of *stratum radiatum*^15,86–89^. Yet, as shown here, spine clustering is a highly dynamic process along CA1 *stratum radiatum* dendrites. We conclude that the resource-rich spines were ready to express lasting LTP by enlarging their own synapses and conferring stability and growth to their neighbors, resulting in robust synaptic spine clusters. The local dendritic resources of SER, polyribosomes, and molecular products of synaptic potentiation (such as elevated calcium or kinase activation) could then tag the entire cluster for growth, preservation, and subsequent plasticity^17,22,23,59^. Future work is needed to determine whether the formation and stabilization of distal spine clusters are also supported by postsynaptic resources.

## Methods

### Physiology

All studies were done in accordance with the approved guidelines of the Institutional Animal Care and Use Committee at University of Texas at Austin^16^. Adult male Long-Evans rats aged 60–61 days old (319–323 g) were anesthetized with halothane and decapitated. Hippocampal slices 400 µm thick were collected from the middle third of the hippocampus (2 slices from 2 animals) and recovered in an interface chamber in artificial cerebrospinal fluid (16.4 mM NaCl, 5.4 mM KCl, 3.2 mM CaCl_2_, 1.6 mM MgSO_4_, 26.2 mM NaHCO_3_, 1.0 mM NaH_2_PO_4_, and 10 mM dextrose) for ~3 hours at 32 °C (Fig. 1A, adapted from Bourne and Harris, 2011). A recording electrode was placed in the middle of *stratum radiatum* in area CA1. Two stimulating electrodes were placed on either side of the recording electrode separated by a distance of 600–800 µm to guarantee stimulation of distinct populations of synapses^4,11^. A stimulus response input-output curve was generated, and the initial slope of the field excitatory potential was measured. The stimulus intensity was set at 75% maximum, above the population spike threshold. This intensity was held constant throughout the experiment. Baseline recordings were collected from each stimulating electrode every 2 minutes (offset by 30 seconds) for ~30 minutes. Theta-burst stimulation (TBS, consisting of 8 trains of 10 bursts at 5 Hz of 4 pulses at 100 Hz at 30 second intervals) was delivered to one stimulating electrode at time 0 to produce LTP (Fig. 1B). Responses to test pulses, alternated between the control and LTP stimulating electrodes, were then monitored for 2 hours. The sites (CA3 or subicular side of the recording electrode) of LTP versus control stimulation were alternated between these experiments.

### Fixation and Processing for EM

The electrodes were removed and hippocampal slices were fully fixed within 1 minute of the last recording by turning the slice, still on its net, into a mixed aldehyde fixative (6% glutaraldehyde and 2% paraformaldehyde in 0.1 M cacodylate buffer with 2 mM CaCl_2_ and 4 mM MgSO_4_) and microwaving the slice in fixative for 10 sec. Slices were kept in fixative overnight at room temperature and then embedded in agarose and vibra-sliced at 70 µm (Leica WT 1000S, Leica, Nussloch, Germany). For each stimulation site, the vibra-slice containing the electrode indentation along with two adjacent vibra-slices were processed for EM through a 1% osmium/1.5% potassium ferrocyanide mixture, 1% osmium alone, dehydrated through graded ethanols (50–100%) and propylene oxide, embedded in LX112, and placed in a 60 °C oven for 48 hours^4^. Three samples were obtained from each experiment spanning the regions beside and beneath the stimulating electrodes. The absolute positions of the 3DEM samples were located ~120 µm away from the stimulating electrode tips, at a depth of ~100 µm from the slice air surface (Fig. 1A).

### Criteria for Experiment and Dendrite Inclusion and Unbiased Analyses

Initially, 70 electrophysiology experiments were performed to establish criteria for saturated LTP and excellent tissue preservation. Then 2 experiments among the last set of 10 were selected for 3DEM analyses, based on the high quality of the ultrastructure and the consistency of the electrophysiological response. Tissue blocks were coded as to experimental condition so that the experimenter was subsequently masked regarding condition. Approximately 200 serial sections were collected from a region located 150–200 µm lateral to the stimulating or control electrode at a depth of 120–150 µm from the air surface of the slice. The sections were mounted on pioloform-coated slot grids (Synaptek, Ted Pella Inc., Redding, CA) and counterstained with ethanolic uranyl acetate and Reynolds lead citrate. The serial sections and a calibration grid (Ted Pella Inc.) were then imaged on a JEOL 1230 transmission electron microscope (Peabody, MA) with a Gatan digital camera.

Serial section images were given a five-letter code, imported into Reconstruct (freely available at synapweb.clm.utexas.edu), and aligned. Section thickness was computed using the cylindrical diameters method: dividing the diameters of longitudinally sectioned mitochondria by the number of sections the mitochondria spanned^90^. All dendrites that spanned the central section of the series were numbered and viewed through serial sections to ascertain their orientation and caliber by measuring the diameter and counting microtubule numbers on sections near the beginning, middle, and end of the series. To be included, dendrites needed to be cross-sectioned for most of their length, as it is difficult to identify spine origins and SER when dendrites are longitudinally sectioned. In addition, the dendrites had to have similar calibers such that spine density in the control condition was not correlated with microtubule number (Fig. 5D). A total of 9 control dendrites (4 from animal 1, 5 from animal 2) and 9 LTP dendrites (5 from animal 1, 4 from animal 2) met these criteria and were used in these analyses.

### Identification of SER branches in the dendritic shafts

To identify points where SER branched, we wrote a simple script in Python that treated individual SER traces created on each section in Reconstruct as vertices. Edges between vertices were identified when the traces overlapped one another on adjacent serial sections. For a trace, the number of SER branching events between two EM sections, b(v), was defined as b(v) = max(deg(v) – 2, 0), where deg(v) is the degree of the vertex v, or the number of edges to which the vertex belonged. Non-branching SER traces existed when the vertex had zero, one, or two neighboring traces on adjacent sections (for deg(v) = 0, 1 or 2) and b(v) was zero for each of these cases to avoid negative numbers. In other words, zero was a disconnected piece of SER, one was the end of a tubule, and two was the middle of a tubule. A vertex with three edges had a single branch, and so on. This metric proceeded linearly such that all SER segments were followed through adjacent serial sections and the number of branch points summed on each section (Fig. 5C).

### Statistical Analyses

Statistical analyses were performed in STATISTICA (TIBCO software Inc., Palo Alto, CA). Chi-squared test of independence was used to investigate changes in the proportions of spines with different SER content. The distributions of synapse areas and spine head diameters were significantly skewed; hence, these data were log_10_ transformed for the reported statistical analyses. Hierarchical nested analyses of variance (hnANOVAs) were performed when many measures (e.g. synaptic areas, head diameters) were obtained from individual dendrites (with dendrite nested in condition and experiment, and experiment nested in condition). When just one or a few measures were obtained from a dendrite (e.g. length, clusters) then full-factorial or one-way ANOVAs were performed controlling for experiment, and the F values with subscripted degrees of freedom (df) presented for condition and error in the figure legends with absolute p values (p < 0.001, minimum reported value). Levene’s test for homogeneity of variance was performed to assure validity of the ANOVA. Analyses of covariance (ANCOVA) were performed while controlling for head diameter (Figs 2G,H and 3C–F) and SER branch number in the synaptic clusters (Fig. 5G,H). Post-hoc analyses were subjected to the Fisher Least Squares Differences (LSD) test and are reported where appropriate in the figure legends. For ease of understanding, the actual mean sizes were plotted as means with SEMs or as true values on the log10 vs log10 plots with associated power trendlines. Analysis of results from each statistical test are reported in the figure legends with significance set to p < 0.05 and asterisks in figures denoting: *p < 0.05, **p < 0.02, ***p < 0.01, and ****p < 0.001. Effect sizes (partial eta-squared, η^2^) were computed (Sum of Squares (SS) factor/(SS factor +SS error)) depending on the specific factor (e.g. LTP vs Control, spine or segment resources) as in the text or legends. In the text, effect sizes are reported as a percentage based on the η^2^ ratio multiplied by 100%.

## Data Availability

The datasets generated during and/or analyzed during the current study are available from the corresponding author on reasonable request.
